# Pasta Consumption Is Linked to Greater Nutrient Intakes and Improved Diet Quality in American Children and Adults, and Beneficial Weight-Related Outcomes Only in Adult Females

**DOI:** 10.3389/fnut.2020.00112

**Published:** 2020-08-07

**Authors:** Yanni Papanikolaou

**Affiliations:** Nutritional Strategies Inc, Paris, ON, Canada

**Keywords:** NHANES, nutrients, pasta, children, adults, diet quality, weight outcomes

## Abstract

The present analyses evaluated associations between pasta consumption, nutrient intakes, and diet quality in U.S. children (2–18 years-old; *N* = 323) and adults (≥19 years-old; *N* = 400) using the US National Health and Nutrition Examination Survey, 2001–2012 dataset. An additional aim included assessing associations with pasta consumption and weight-related outcomes in adults. Consumption of dry, domestic, and imported wheat pasta/noodles without eggs defined pasta consumers. Energy intake was similar when comparing pasta consumers vs. non-consumers of pasta. Pasta consumers had increased daily intake of dietary fiber (16 ± 0.6 vs. 13 ± 0.1 g/d, *p* < 0.0001; 21 ± 0.8 vs. 16 ± 0.1 g/d, *p* < 0.0001), folate, DFE (701 ± 30 vs. 528 ± 5 μg/d, *p* < 0.0001; 733 ± 42 vs. 546 ± 4 μg/d, *p* < 0.0001), iron (16 ± 0.5 vs. 14 ± 0.1 mg/d, *p* = 0.01; 18 ± 0.9 vs. 16 ± 0.1 mg/d, *p* = 0.01), magnesium (249 ± 7 vs. 231 ± mg/d, *p* = 0.006; 327 ± 12 vs. 297 ± 2 mg/d, *p* < 0.02), and vitamin E as α-tocopherol (7 ± 0.4 vs. 6 ± 0.05 mg/d, *p* = 0.012; 10.0 ± 0.4 vs. 7.7 ± 0.1 mg/d, *p* < 0.0001), when compared to non-consumers of pasta, in children and adults, respectively. Daily intakes for potassium, calcium, vitamin A and vitamin D were similar when comparing pasta consumers to non-consumers of pasta. Adult pasta consumers had reduced added sugar and saturated fat intake, while no differences were observed for sodium intake vs. non-pasta consumption. Pasta consumption in children was associated with lower saturated fat, with no differences seen in added sugar and sodium intake. Pasta consumption was associated with an improved diet quality relative to non-pasta consumption (children: 48.6 ± 1.0 vs. 45.6 ± 0.2, *p* = 0.002; adult: 51.0 ± 0.9 vs. 48.9 ± 0.2, *p* = 0.024). No associations were observed when evaluating pasta consumption in all adults, however, gender-specific analysis revealed reduced body mass index, waist circumference, and body weight in females aged 19–50 years when compared to no pasta consumption. Overall, pasta consumption was associated with a better diet quality, improved nutrient intakes and lower intake of nutrients to limit relative to non-pasta consumption in Americans.

## Introduction

Pasta remains a routine food consumed by Americans, however, recent shifts in consumer perceptions around carbohydrate intake and nutrition (1) are likely contributing to a decline in pasta consumption in the US. Indeed, the Prospective Urban Rural Epidemiology (PURE) analysis, involving a large global cohort identified that increased carbohydrate intake was linked to a higher risk of total mortality, but not cardiovascular-related mortality, however, type of carbohydrate was not differentiated (2). The popularity of the low-carbohydrate diets has led global media to report on “good and bad carbs,” indicating that certain carbohydrates, relative to others, have inherent beneficial attributes to health (3). Consumption trends in American adults using data from the Framingham Heart Study has shown a significant reduction in the number of weekly servings of several carbohydrate-based foods, including pasta, yeast breads, rice, cooked grains and indulgent grains (4). The 2015 Dietary Guidelines for Americans (2015-DGA) promote an increased consumption of whole grains (i.e., at least half of which are whole grains), while concurrently advising Americans to limit refined grains in the diet (5). Alongside grain recommendations, the 2015-DGA has identified several nutrients under-consumed in current food patterns in comparison to required levels set by the Institute of Medicine (IOM)—the designated shortfall nutrients are: vitamin A, vitamin D, vitamin E, vitamin C, folate, calcium, magnesium, fiber, and potassium and for adolescent and premenopausal females, iron has also been included as a shortfall nutrient. The 2015-DGA also characterized fiber, potassium, calcium, and vitamin D as “nutrients of public health concern” since under-consumption has been linked to adverse health outcomes (5).

Recent analyses from the National Health and Nutrition Examination Survey (NHANES) have demonstrated the nutrient density contribution of select grain foods in American dietary patterns. Nonetheless, some grain-based foods may contribute greater levels of nutrients to limit, including added saturated fat, added sugar, and sodium. However, in contrast, many grain foods provide positive nutrition to the North American diet and have been shown to be important vehicles of under-consumed nutrients (6–9). A cluster analyses using data from NHANES 2005–2010 reported that various grain food patterns were positively associated with nutrient intakes and diet quality (10, 11), including improved intake of several shortfall nutrients and nutrients of public health concern as identified by the 2015-DGA (5). Specifically, a dietary pattern consisting mainly of pasta, cooked cereals and rice in adults, was linked to greater daily fiber and lower added sugar intake, in comparison to a control group avoiding grain foods consumption. Similarly, children consuming several grain-based dietary patterns, of which included both refined and whole grains, had improved intakes of shortfall nutrients and/or nutrients of concern, including fiber, magnesium, iron, folate, and vitamin D, relative to children with dietary patterns that limited grains (10, 11). Data from over 14,000 adults in the Italian Moli-sani cohort showed that pasta consumption was associated with better adhesion to the Mediterranean diet in both males and females. Further comparisons that included adults from the Moli-sani cohort and Italian Nutrition and Health Survey (*n* = 8,964) substantiated a negative association between pasta intake and indexes of obesity status and prevalence of overweight and obesity (12). A recent NHANES cluster analyses found that pasta consumption patterns in adults were associated with diet quality, such that type of pasta and food groups accompanying pasta dishes, could contribute to diet quality outcome (13). When cooked for shorter durations, pasta acts as a slowly-absorbed carbohydrate (14). Consuming slowly absorbed carbohydrates have been shown to have beneficial metabolic outcomes and include reduced postprandial glucose and insulin responses, which may help moderate rate of digestion, increase satiety and reduce hunger (14–17). Nonetheless, there are limited published data involving American children when evaluating pasta consumption with nutrient and health outcomes. Additionally, there are limited data examining associations between pasta foods and weight-related variables in adults.

The objective of the present analyses was to determine associations between pasta consumption, nutrient intakes, and diet quality in children and adults compared to non-consumption of pasta using data from NHANES 2001–2012. Further, the present study aims to assess associations with tertile pasta consumption and weight-related outcomes in adults.

## Experimental Section

NHANES datasets are collected by the National Center for Health Statistics of the Centers for Disease Control and Prevention. NHANES collects data from free-living/non-institutionalized Americans to generate a cross-sectional survey that is nationally representative. Written informed consent and ethical review of NHANES has been previously documented and granted approval by the National Center for Health Statistics-Research Ethics Review Board and the present study did not require supplementary ethics review (18, 19). Six NHANES datasets (2001–2002; 2003–2004; 2005–2006; 2007–2008; 2009–2010; 2011–2012) were amalgamated for the current analyses (19–21). Data for nutrient intakes were sourced from the Food and Nutrient Database for Dietary Studies (FNDDS), United States Department of Agriculture (USDA) (22, 23). FNDDS provide the nutrient values for foods and beverages reported in What We Eat in America (WWEIA), the dietary intake component of NHANES for each data release. The WWEIA Food Categories provide an application to analyze food and beverages as consumed in the American diet and the classification scheme includes 150 unique categories with 15 main food groups and 46 subcategories of foods. WWEIA food categories have been previously published by USDA (20).

In the present analyses, 323 children aged 2–18 years comprised the study sample for pasta consumers, representing a population sample of 1,568,241 Americans. For adults ≥19 years of age, the combined study adult sample included 400 male and female pasta consumers, which represented a population sample of 4,357,392 Americans. All participant data from What We Eat in American (WWEIA), the dietary intake component of NHANES, was verified for reliability and completeness (i.e., 24-h dietary intakes). All reported pregnant females at the time of NHANES data collection were omitted from the analyses.

### Methods and Statistical Analysis

All statistical analyses in the present study were performed using SAS (version 9.2, SAS Institute, Cary, NC, USA) and SUDAAN software (version 11.0, Research Triangle Park, NC, USA). Survey weights were used to generate nationally representative estimates for US adults, which were adjusted for the complex sample design of NHANES. Pasta consumption was defined as all dry domestic and imported pasta/noodle varieties made with only wheat and no egg. Estimation of pasta intakes were assessed using 16 USDA food codes for pasta (Table 1).

**Table 1:** USDA food codes for pasta foods and corresponding food descriptions (12, 18).

**Food Codes**	**Description**
56101000	Macaroni, cooked, NS as to fat added in cooking
56101010	Macaroni, cooked, fat not added in cooking
56101030	Macaroni, cooked, fat added in cooking
56102000	Macaroni, whole wheat, cooked, NS as to fat added in cooking
56102010	Macaroni, whole wheat, cooked, fat not added in cooking
56102020	Macaroni, whole wheat, cooked, fat added in cooking
56103010	Macaroni, cooked, spinach, fat not added in cooking
56104020	Macaroni, cooked, vegetable, fat added in cooking
56113010	Noodles, cooked, whole wheat, fat not added in cooking
56114000	Noodles, cooked, spinach, fat not added in cooking
56130000	Spaghetti, cooked, NS as to fat added in cooking
56130010	Spaghetti, cooked, fat not added in cooking
56131000	Spaghetti, cooked, fat added in cooking
56132990	Spaghetti, cooked, whole wheat, NS as to fat added in cooking
56133000	Spaghetti, cooked, whole wheat, fat not added in cooking
56133010	Spaghetti, cooked, whole wheat, fat added in cooking

Regression analyses of intakes from the first-day dietary recall (i.e., in person interview) were completed to examine differences between pasta consumers and non-consumers of pasta, with the exclusion of mixed dishes containing pasta. Adjusted least square mean ± standard error (SE) for energy and nutrients were determined for pasta consumers and non-consumers of pasta with various sets of covariates. Covariates for analyses of energy intake, Healthy Eating Index (HEI)-2010 and HEI sub-components were age, gender, ethnicity, poverty income ratio, physical activity (sedentary, moderate or vigorous based on questionnaire responses), current smoking status, and alcohol intake. Nutrient intakes were also adjusted for energy intakes. The HEI designates a measure of diet quality and conformance to US dietary guidance and has been predominantly used historically to monitor dietary practices of Americans (23). Data are presented as means ± SE and a *p*-value of < 0.05 was deemed as significant. The analyses also determined least square means ± standard error (SE) for four levels of pasta intake, including zero intake (i.e., no pasta consumption) and three tertile ranges for pasta consumers. The tertiles used were based on the pasta intakes for the population (i.e., age group and gender) for each regression analysis. Similarly, all data are presented as means ± SE and a *p*-value of < 0.05 for group trend was interpreted as significant for all calculations.

## Results

### Energy and Nutrient Intakes in Pasta Consumers Compared to Non-consumers of Pasta

Tables 2, 3 lists the demographic data of the study population. In general, with the exception of pasta consumption, smoking and PIR < 1.35% status, demographics between pasta consumers and non-consumers of pasta were similar in the adult population. In children, with the exemption of pasta consumption, demographics between consumers and non-consumers were not significantly different.

**Table 2:** Demographic characteristics of adults of different age groups by pasta consumption.

**Age**	**Range**		**Pasta non-consumers**		**Pasta consumers**		***P***
		**Variable**	**Mean**	**SE**	**Mean**	**SE**	
2–18 Years		Age	10.07	0.07	9.27	0.36	0.0289
		Gender = Male (%)	50.64	0.59	51.37	4.45	0.8714
		PIR < 1.35 (%)	33.12	1.02	24.54	4.94	0.0890
		1.35 < = PIR < = 1.85 (%)	11.38	0.51	11.74	3.99	0.9297
		PIR > 1.85 (%)	55.49	1.13	63.73	5.74	0.1596
		Phys Activity = Sedentary (%)	12.63	0.39	8.58	2.30	0.0815
		Phys Activity = Moderate (%)	19.83	0.52	23.22	4.18	0.4206
		Phys Activity = Vigorous (%)	67.53	0.61	68.20	3.30	0.8426
		Smoking current (%)	2.70	0.27	1.28	0.65	0.0413
		Overweight (%)	15.30	0.43	15.23	2.52	0.9771
		Obese (%)	16.21	0.51	18.55	3.92	0.5548
		Pasta (grams)	0.00	0.00	167.63	8.46	0.0000
		Sample N	20,006		323		
		Population N	67,755,035		1,568,241		

*Data are from NHANES 2009–2012; PIR, poverty income ratio; SE, standard error; all statistical analyses are based on the sample N*.

**Table 3:** Demographic characteristics of adults of different age groups by pasta consumption.

**Age**	**Range**		**Pasta non-consumers**		**Pasta consumers**		***P***
		**Variable**	**Mean**	**SE**	**Mean**	**SE**	
19 to 50		Age	34.84	0.18	33.92	0.87	0.2957
		Gender = Male (%)	51.32	0.45	53.08	4.94	0.7214
		PIR < 1.35 (%)	24.95	0.97	18.13	2.87	0.0245
		1.35 < = PIR < = 1.85 (%)	10.24	0.37	12.43	3.41	0.5237
		PIR > 1.85 (%)	64.81	1.05	69.44	4.24	0.2894
		Phys Activity = Sedentary (%)	21.87	0.66	16.91	3.73	0.1898
		Phys Activity = Moderate (%)	30.80	0.62	28.23	3.64	0.4859
		Phys Activity = Vigorous (%)	47.33	0.88	54.87	4.78	0.1211
		Smoking current (%)	25.07	0.80	18.79	3.48	0.0787
		Overweight (%)	31.13	0.62	32.89	3.93	0.6578
		Obese (%)	32.11	0.67	27.21	3.75	0.1987
		Pasta (grams)	0.00	0.00	217.83	25.52	<0.0001
		Sample N	15,717		218		
		Population N	124,445,536		2,670,120		
51 to 99		Age	64.07	0.14	62.40	0.93	0.0762
		Gender = Male (%)	46.19	0.45	40.71	4.52	0.2281
		PIR < 1.35 (%)	17.76	0.73	14.71	3.52	0.3951
		1.35 ≤ PIR ≤ 1.85 (%)	10.14	0.39	6.02	2.28	0.0741
		PIR > 1.85 (%)	72.10	0.91	79.28	3.98	0.0789
		Phys Activity = Sedentary (%)	36.12	0.74	39.70	5.45	0.5148
		Phys Activity = Moderate (%)	41.34	0.57	42.91	5.05	0.7576
		Phys Activity = Vigorous (%)	22.55	0.76	17.39	3.00	0.0962
		Smoking Current (%)	16.79	0.45	12.21	2.93	0.1225
		Overweight (%)	36.75	0.63	45.06	5.34	0.1225
		Obese (%)	35.90	0.67	36.68	6.13	0.8993
		Pasta (grams)	0.00	0.00	189.61	13.79	<0.0001
		Sample N	13,567		182		
		Population N	83,468,702		1,687,272		
19 to 99		Age	46.58	0.27	44.95	1.05	0.1317
		Gender = Male (%)	49.26	0.34	48.29	3.20	0.7647
		PIR < 1.35 (%)	22.09	0.71	16.81	2.44	0.0382
		1.35 ≤ PIR ≤ 1.85 (%)	10.20	0.29	9.95	2.29	0.9146
		PIR > 1.85 (%)	67.71	0.83	73.24	3.32	0.1065
		Phys Activity = Sedentary (%)	27.59	0.57	25.70	3.22	0.5641
		Phys Activity = Moderate (%)	35.03	0.44	33.89	2.92	0.6994
		Phys Activity = Vigorous (%)	37.38	0.71	40.40	3.88	0.4432
		Smoking current (%)	21.74	0.58	16.24	2.38	0.0246
		Overweight (%)	33.37	0.51	37.61	3.01	0.1654
		Obese (%)	33.62	0.52	30.88	3.28	0.4086
		Pasta (grams)	0.00	0.00	206.90	17.11	<0.0001
		Sample N	29,284		400		
		Population N	207,914,238		4,357,392		

*Data are from NHANES 2009–2012; PIR, poverty income ratio; SE, standard error; All statistical analyses are based on the sample N*.

### Energy and Nutrient Intakes

#### Children

Significant associations were not observed between pasta consumers and non-consumers of pasta for daily energy intake (2029 ± 51.7 vs. 1985 ± 8.9 kcal/day, *p* = 0.4075) (Table 4). When considering 2015-DGA nutrients of concern, children consuming pasta had increased intakes of dietary fiber (15.6 ± 0.6 vs. 13.1 ± 0.1 g/day, *p* < 0.0001) relative to non-consumers of pasta, with no significant associations for vitamin D, calcium and potassium. Children consuming pasta also had greater intake of several 2015–2020 Dietary Guideline shortfall nutrients. Specifically, pasta consumers had higher intakes of magnesium (249.4 ± 6.9 vs. 230.9 ± 1.4 mg/day; *p* = 0.0063), iron (15.8 ± 0.5 vs. 14.4 ± 0.1 mg/day; *p* = 0.0144), dietary folate, DFE (700.7 ± 30.0 vs. 528.0 ± 5.0 μg/day; *p* < 0.0001), and vitamin E, alpha-tocopherol (7.1 ± 0.4 vs. 6.0 ± 0.1 mg/day; *p* = 0.0116) relative to non-consumers of pasta.

**Table 4:** Adjusted energy and nutrient intakes in Pasta Consumers vs. Non-consumers of Pasta^a^.

**Energy/nutrients**	**Pasta non-consumers**		**Pasta consumers**		**Beta**	***P***
	**LSM**	**SE**	**LSM**	**SE**		
Energy (kcal)	1985.22	8.91	2028.78	51.71	43.56	0.4075
Carbohydrate (gm)	267.31	1.23	295.08	7.95	27.77	0.0007
Protein (gm)	69.51	0.42	68.61	1.99	−0.89	0.6512
Total fat (g)	73.07	0.43	66.48	2.37	−6.60	0.0087
Total monounsaturated fatty acids (g)	26.58	0.17	23.54	0.93	−3.04	0.0021
Total polyunsaturated fatty acids (g)	14.68	0.11	14.18	0.61	−0.50	0.4168
Total saturated fatty acids (g)	25.70	0.16	23.03	0.93	−2.67	0.0071
Cholesterol (mg)	221.26	2.02	192.72	10.74	−28.53	0.0096
Added sugars (tsp eq)	20.62	0.19	18.82	1.03	−1.81	0.1053
Total sugars (g)	135.31	0.85	136.99	4.61	1.68	0.7269
Dietary fiber (g)	13.07	0.10	15.58	0.56	2.52	<0.0001
Calcium (mg)	1019.48	7.94	1023.42	44.54	3.94	0.9301
Iron (mg)	14.37	0.10	15.77	0.54	1.40	0.0144
Folate, DFE (mcg)	528.04	5.02	700.69	29.93	172.65	<0.0001
Magnesium (mg)	230.90	1.37	249.42	6.93	18.52	0.0063
Niacin (mg)	20.90	0.16	21.35	0.60	0.46	0.4393
Phosphorus (mg)	1260.86	7.46	1221.66	39.84	−39.20	0.3167
Potassium (mg)	2227.46	15.66	2279.11	76.66	51.65	0.4843
Riboflavin (Vitamin B2) (mg)	2.08	0.02	2.22	0.06	0.14	0.0224
Selenium (mcg)	92.52	0.62	114.18	3.82	21.66	<0.0001
Sodium (mg)	3129.95	20.87	3096.88	92.95	−33.07	0.7205
Thiamin (Vitamin B1) (mg)	1.55	0.01	1.75	0.06	0.19	0.0015
Vitamin A, RAE (mcg)	584.30	6.59	612.96	26.84	28.66	0.3053
Vitamin B12 (mcg)	5.04	0.05	4.97	0.31	−0.08	0.8052
Vitamin B6 (mg)	1.70	0.02	1.66	0.11	−0.03	0.7576
Vitamin C (mg)	82.82	1.26	87.79	5.07	4.97	0.3366
Vitamin D (D2 + D3) (mcg)	5.92	0.07	6.42	0.30	0.50	0.1026
Vitamin E as alpha-tocopherol (mg)	6.03	0.05	7.06	0.40	1.03	0.0116
Vitamin K (mcg)	55.29	0.95	61.39	4.17	6.10	0.1539
Zinc (mg)	10.59	0.07	10.80	0.39	0.21	0.5858

a *Data are for children, combined gender, aged 2–18 years-old; Day 1 intakes; tsp eq, teaspoon equivalents; DFE, dietary folate equivalents. LSM, least square mean; SE, standard error*.

Assessment of 2015-DGA nutrients to limit revealed no differences in intake of added sugars, but lower intake of saturated fat (23.0 ± 0.9 vs. 25.7 ± 0.2 g/day; *p* = 0.0071) in pasta consumers compared to non-consumers of pasta, with no significant differences observed in sodium intakes. In addition, no significant associations were seen with total sugars and total fat consumption. Total cholesterol intake was also lower in children consuming pasta compared to non-pasta consumers. All nutrient intakes in pasta consumers relative to non-consumers of pasta are listed in Table 4.

#### Adults

Significant differences were not seen between pasta consumers and non-consumers of pasta for daily energy intake (2240 ± 63 vs. 2180 ± 8 kcal/day, *p* = 0.3546) (Table 5). When considering 2015-DGA nutrients of concern, pasta consumers had greater intakes of dietary fiber (20.6 ± 0.8 vs. 16.4 ± 0.1 g/day, *p* < 0.0001) relative to non-consumers of pasta, with no significant differences seen for vitamin D, calcium and potassium. Pasta consumers had greater intake of several 2015–2020 Dietary Guideline shortfall nutrients relative to non-consumers—pasta consumers relative to non-consumers of pasta had higher intakes of magnesium (326.7 ± 12.1 vs. 296.8 ± 2.0 mg/day; *p* = 0.016), iron (17.9 ± 0.9 vs. 15.6 ± 0.1 mg/day; *p* = 0.012), dietary folate, DFE (732.6 ± 41.6 vs. 546.0 ± 4.2 μg/day; *p* < 0.0001), vitamin C (107.1 ± 8.5 vs. 87.4 ± 1.2 mg/day; *p* = 0.019), and vitamin E, alpha-tocopherol (10.0 ± 0.4 vs. 7.7 ± 0.1 mg/day; *p* < 0.0001).

**Table 5:** Adjusted energy and nutrient intakes in Pasta Consumers vs. Non-consumers of Pasta^a^.

**Outcome**	**Pasta non-consumers**		**Pasta consumers**		**Beta**	***P***
	**LSM**	**SE**	**LSM**	**SE**		
Energy (kcal)	2179.96	8.39	2239.69	63.27	59.73	0.3546
Protein (g)	83.09	0.37	84.58	3.10	1.49	0.6386
Carbohydrate (g)	264.53	1.17	290.59	9.73	26.07	0.0094
Total fat (g)	82.31	0.44	77.51	2.70	−4.81	0.0839
Total monounsaturated fatty acids (g)	30.16	0.17	27.81	1.05	−2.35	0.0321
Total polyunsaturated fatty acids (g)	17.83	0.11	17.77	0.68	−0.06	0.9303
Total saturated fatty acids (g)	26.99	0.17	24.60	1.17	−2.39	0.0437
Cholesterol (mg)	286.71	2.01	268.96	15.89	−17.74	0.2741
Dietary fiber (g)	16.38	0.14	20.63	0.81	4.25	<0.0001
Total sugars (g)	122.18	0.84	116.95	5.26	−5.23	0.3261
Calcium (mg)	943.42	6.39	916.54	60.90	−26.87	0.6641
Folate, DFE (mcg)	545.97	4.15	732.59	41.64	186.62	<0.0001
Iron (mg)	15.59	0.09	17.85	0.87	2.26	0.0118
Magnesium (mg)	296.76	1.99	326.67	12.09	29.91	0.0161
Niacin (mg)	25.16	0.14	26.56	1.20	1.40	0.2484
Phosphorus (mg)	1369.28	6.51	1377.99	54.71	8.71	0.8768
Potassium (mg)	2736.64	14.26	2874.66	107.42	138.01	0.2085
Riboflavin (Vitamin B2) (mg)	2.22	0.01	2.29	0.09	0.08	0.4136
Selenium (mcg)	110.85	0.60	142.57	4.97	31.72	<0.0001
Sodium (mg)	3617.50	15.28	3849.58	137.79	232.08	0.1038
Thiamin (Vitamin B1) (mg)	1.65	0.01	1.91	0.07	0.26	0.0012
Vitamin A, RAE (mcg)	629.32	9.59	677.12	51.64	47.80	0.3747
Vitamin B12 (mcg)	5.42	0.07	4.86	0.31	−0.56	0.0688
Vitamin B6 (mg)	2.02	0.01	2.07	0.12	0.05	0.6675
Vitamin C (mg)	87.35	1.18	107.14	8.48	19.79	0.0192
Vitamin D (D2 + D3) (mcg)	4.71	0.06	4.60	0.44	−0.10	0.8220
Vitamin E as alpha-tocopherol (mg)	7.71	0.07	9.97	0.44	2.26	<0.0001
Vitamin K (mcg)	102.39	2.06	132.67	18.33	30.28	0.1168
Zinc (mg)	12.09	0.08	12.35	0.56	0.27	0.6319

a *Data are for adults, combined gender, aged ≥19 years-old; Day 1 intakes; tsp eq, teaspoon equivalents; DFE, dietary folate equivalents; LSM, least square mean; SE, standard error*.

Assessment of 2015-DGA nutrients to limit revealed lower added sugars (16.1 ± 0.8 vs. 19.3 ± 0.2 tsp eq/day; *p* = 0.0003) and saturated fat (24.6 ± 1.2 vs. 27.0 ± 0.2 g/day; *p* = 0.04) intakes in pasta consumers compared to non-consumers of pasta, with no significant differences observed in sodium intakes between the two groups. In addition, no significant associations were seen with total sugars and total fat consumption. All nutrient intakes in pasta consumers relative to non-consumers of pasta are listed in Table 5.

### Diet Quality in Pasta Consumers Compared to Non-consumers of Pasta

Diet quality, as measured by USDA's HEI-2010 is depicted in Tables 6, 7.

**Table 6:** Adjusted healthy eating Index scores in Pasta Consumers vs. Non-consumers of Pasta^a^.

**HEI total and sub-component variables**	**Pasta non-consumers**		**Pasta consumers**		***P***
	**LSM**	**SE**	**LSM**	**SE**	
HEI 2010 component 1 (Total vegetables)	2.10	0.02	2.19	0.12	0.4341
HEI 2010 component 2 (Greens and beans)	0.59	0.02	0.69	0.13	0.4857
HEI 2010 component 3 (Total fruit)	2.49	0.04	2.90	0.20	0.0394
HEI 2010 component 4 (Whole fruit)	2.12	0.04	2.55	0.21	0.0420
HEI 2010 component 5 (Whole grains)	1.91	0.04	2.28	0.31	0.2394
HEI 2010 component 6 (Dairy)	6.97	0.04	6.89	0.23	0.7252
HEI 2010 component 7 (Total protein foods)	3.54	0.02	2.81	0.13	<0.0001
HEI 2010 component 8 (Seafood and plant protein)	1.41	0.03	1.38	0.19	0.8890
HEI 2010 component 9 (Fatty acid ratio)	3.79	0.04	4.15	0.31	0.2396
HEI 2010 component 10 (Sodium)	5.05	0.05	5.40	0.25	0.1674
HEI 2010 component 11 (Refined grains)	5.30	0.05	3.98	0.31	0.0001
HEI 2010 component 12 (SoFAAS calories)	10.26	0.10	13.42	0.36	<0.0001
HEI 2010 total score	45.55	0.22	48.64	0.99	0.0021

a *Data are for children, combined gender, 2–18 years-old (N = 323); Day 1 intakes; SoFAAS, solid fats, alcohol and added sugars; LSM, least square mean; SE, standard error*.

**Table 7:** Adjusted healthy eating Index-2010 scores in Pasta Consumers vs. Non-Consumers of Pasta^a^.

**HEI total and sub-component variables**	**Pasta non-consumers**		**Pasta consumers**		***P***
	**LSM**	**SE**	**LSM**	**SE**	
HEI 2010 component 1 (Total vegetables)	3.03	0.02	3.26	0.09	0.0237
HEI 2010 component 2 (Greens and beans)	1.22	0.02	1.35	0.16	0.4242
HEI 2010 component 3 (Total fruit)	2.10	0.03	2.28	0.17	0.2958
HEI 2010 component 4 (Whole fruit)	2.01	0.03	2.17	0.19	0.4032
HEI 2010 component 5 (Whole grains)	2.28	0.04	2.78	0.26	0.0533
HEI 2010 component 6 (Dairy)	5.07	0.04	4.57	0.27	0.0656
HEI 2010 component 7 (Total protein foods)	4.18	0.01	3.75	0.12	0.0007
HEI 2010 component 8 (Seafood and plant protein)	1.96	0.02	1.93	0.12	0.7998
HEI 2010 component 9 (Fatty acid ratio)	4.95	0.04	5.68	0.27	0.0059
HEI 2010 component 10 (Sodium)	4.28	0.03	3.81	0.25	0.0573
HEI 2010 component 11 (Refined grains)	6.17	0.04	4.90	0.26	<0.0001
HEI 2010 component 12 (SoFAAS calories)	11.61	0.09	14.54	0.34	<0.0001
HEI 2010 total score	48.88	0.22	51.03	0.94	0.0240

a *Data are for adults, combined gender, aged ≥19 years-old (N = 400); Day 1 intakes; SoFAAS, solid fats, alcohol and added sugars; LSM, least square mean; SE, standard error*.

Pasta consumption in children was associated with a significantly improved total diet quality score (48.64 ± 0.99 vs. 45.55 ± 0.22, *p* = 0.0021). Total fruit, whole fruit intake was greater in pasta consumers relative to non-consumers of pasta. Calories from solid fats, alcohol and added sugar was also higher in pasta consumers vs. non-consumers of pasta. Total protein foods and refined grain intake was significantly lower in pasta consumers compared to non-consumers of pasta. No associations were observed with other sub-component variables, including sodium, fatty acid ratio, whole grains, and vegetables.

Adults who consumed pasta had a better HEI-2010 score relative to non-consumers of pasta (48.64 ± 0.99 vs. 45.55 ± 0.22, *p* = 0.0021). Examination of the subcomponents of HEI-2010 (Table 7) revealed significant associations, such that pasta consumers had a significantly greater score for total vegetables (*p* = 0.0237) suggesting that pasta consumption may be a vehicle for delivery of vegetables in the diet. A significantly lower score was seen with total protein foods in pasta consumers compared to non-consumers of pasta, which may reflect an opportunity to increase lean protein foods with pasta consumption, including lean meats, dairy, plant, and seafood options. Pasta consumers also had higher scores for fatty acid ratio, solid fats, alcohol and added sugars (SoFAAS) and no differences with sodium scores relative to non-consumers.

### Weight-Related Outcome Measurements in Adult Male and Female Pasta Consumers Compared to Non-consumers of Pasta

As a previous NHANES analysis (8), showed that females consumed more pasta dishes relative to males, but did not examine associations with weight-related outcomes, the current study examined relationships between pasta tertile consumption and skinfold measurements, waist circumference, body weight, and body mass index (BMI) (Tables 8–10). No associations were seen when including all genders ≥19 years-old and older adults, 51–99 years-old. However, pasta intake across tertile ranges showed that pasta consumption in females aged 19–50 years-old, was associated with reduced waist circumference, body weight, and BMI (Table 9). No significant associations were observed when assessing pasta consumption and triceps and triceps + subscapular skinfold measurements. Similarly, in children, no significant associations were seen with any weight-related parameter examined (data not shown).

**Table 8:** Adjusted mean weight-related health measurements in males and females by Tertile of Pasta Consumption vs. No Pasta Consumption^a^.

**Outcome**	**Gender**	**Q1-No pasta**		**Q2**		**Q3**		**Q4**		***P***
		**LSM**	**SE**	**LSM**	**SE**	**LSM**	**SE**	**LSM**	**SE**	
Triceps skinfold (mm)	All	19.18	0.09	19.57	0.95	18.72	0.70	18.92	0.79	0.6512
Triceps skinfold (mm)	Male	14.78	0.11	15.38	0.85	15.24	1.33	13.66	1.15	0.6388
Triceps skinfold (mm)	Female	23.77	0.11	21.36	1.84	24.92	1.43	23.47	0.86	0.9786
Triceps+subscapular skinfold (mm)	All	38.17	0.19	38.91	2.03	38.08	1.34	38.71	2.08	0.7621
Triceps+subscapular skinfold (mm)	Male	33.05	0.20	36.03	1.89	34.86	3.55	31.30	2.10	0.9305
Triceps+subscapular skinfold (mm)	Female	43.49	0.25	38.17	3.91	45.41	2.71	44.26	2.13	0.6931
Waist circumference (cm)	All	97.49	0.19	100.59	2.62	96.65	1.49	97.35	1.46	0.8811
Waist circumference (cm)	Male	100.36	0.20	104.21	1.99	101.77	1.91	100.54	2.30	0.4022
Waist circumference (cm)	Female	94.69	0.25	96.16	3.41	95.94	3.10	92.37	2.25	0.7599
Weight (kg)	All	81.51	0.22	86.99	3.35	79.54	1.97	81.47	1.95	0.8711
Weight (kg)	Male	88.11	0.25	92.84	2.84	89.03	2.20	87.69	3.34	0.6729
Weight (kg)	Female	75.09	0.28	77.03	3.82	77.32	4.60	73.66	3.13	0.8944
Body Mass Index (kg/m^2^)	All	28.44	0.08	30.37	1.28	27.24	0.67	28.36	0.54	0.7579
Body Mass Index (kg/m^2^)	Male	28.33	0.08	29.58	0.86	28.39	0.63	27.98	0.82	0.9730
Body Mass Index (kg/m^2^)	Female	28.54	0.10	29.06	1.42	29.46	1.89	27.74	1.08	0.9915

a *Data are for adults, combined gender, aged ≥19 years-old (N = 400); Day 1 intakes; SE, standard error*.

**Table 9:** Adjusted mean weight-related health measurements in males and females by Tertile of Pasta Consumption vs. No Pasta Consumption^a^.

**Outcome**	**Gender**	**Q1-no pasta**		**Q2**		**Q3**		**Q4**		***P***
		**LSM**	**SE**	**LSM**	**SE**	**LSM**	**SE**	**LSM**	**SE**	
Triceps skinfold (mm)	All	18.71	0.11	19.63	1.39	18.03	0.97	18.96	1.24	0.9648
Triceps skinfold (mm)	Male	14.43	0.12	16.27	1.22	14.32	1.93	13.64	1.54	0.8840
Triceps skinfold (mm)	Female	23.58	0.17	21.11	2.67	25.42	2.06	22.91	1.26	0.9939
Triceps+subscapular skinfold (mm)	All	37.19	0.25	38.85	2.91	37.61	1.79	39.85	2.92	0.2907
Triceps+subscapular skinfold (mm)	Male	32.17	0.26	36.65	2.46	35.52	4.43	31.24	3.00	0.5470
Triceps+subscapular skinfold (mm)	Female	42.98	0.38	37.06	5.61	45.92	4.08	45.43	2.99	0.4344
Waist circumference (cm)	All	95.17	0.23	95.83	2.45	91.34	1.25	97.33	2.21	0.9737
Waist circumference (cm)	Male	97.66	0.24	101.25	3.16	98.94	2.96	101.07	3.02	0.1737
Waist circumference (cm)	Female	92.52	0.35	91.84	4.38	89.34	2.05	86.99	2.22	0.0088
Weight (kg)	All	81.71	0.27	85.05	2.94	76.59	1.56	83.86	3.01	0.9719
Weight (kg)	Male	87.91	0.32	93.45	4.36	88.57	3.59	91.04	4.31	0.3536
Weight (kg)	Female	75.16	0.39	76.57	5.21	72.86	2.51	68.10	2.60	0.0269
Body Mass Index (kg/m**2)	All	28.13	0.10	29.17	1.03	26.04	0.46	28.60	0.86	0.6036
Body Mass Index (kg/m**2)	Male	28.05	0.10	29.49	1.16	27.88	1.02	28.65	1.16	0.5453
Body Mass Index (kg/m**2)	Female	28.21	0.15	28.87	2.02	27.01	1.10	25.96	0.91	0.0395

a *Data are for adults, combined gender, aged 19–50 years-old (N = 218, Males = 116, Females = 102) pasta consumers; Day 1 intakes; SE, standard error*.

**Table 10:** Adjusted mean weight-related health measurements in males and females by Tertile of Pasta Consumption vs. No Pasta Consumption^a^.

**Outcome**	**Gender**	**Q1-No pasta**		**Q2**		**Q3**		**Q4**		***P***
		**LSM**	**SE**	**LSM**	**SE**	**LSM**	**SE**	**LSM**	**SE**	
Triceps skinfold (mm)	All	19.89	0.11	19.57	0.93	20.13	1.24	18.52	0.81	0.2392
Triceps skinfold (mm)	Male	15.37	0.16	15.98	1.09	15.07	2.30	13.52	0.90	0.2977
Triceps skinfold (mm)	Female	24.04	0.13	23.26	1.44	24.59	1.12	23.27	1.33	0.6677
Triceps+subscapular skinfold (mm)	All	39.69	0.17	38.98	1.59	40.51	1.58	35.83	1.31	0.0635
Triceps+subscapular skinfold (mm)	Male	34.61	0.25	36.63	2.25	32.59	2.85	31.78	1.50	0.1846
Triceps+subscapular skinfold (mm)	Female	44.18	0.26	41.02	1.96	46.52	1.51	40.99	1.89	0.2830
Waist circumference (cm)	All	101.04	0.22	107.35	5.11	103.17	2.27	99.53	2.50	0.5937
Waist circumference (cm)	Male	104.94	0.28	108.66	2.33	109.21	2.36	97.81	1.73	0.2945
Waist circumference (cm)	Female	97.69	0.27	108.66	6.82	97.81	3.48	98.30	3.67	0.4686
Weight (kg)	All	81.21	0.28	89.84	6.47	80.87	3.02	81.13	3.47	0.5971
Weight (kg)	Male	88.43	0.38	91.79	2.74	93.50	3.65	79.64	2.18	0.3157
Weight (kg)	Female	75.01	0.34	88.50	9.85	72.37	3.71	78.51	4.83	0.3851
Body Mass Index (kg/m^2^)	All	28.91	0.10	32.29	2.60	28.31	0.88	28.73	1.13	0.7761
Body Mass Index (kg/m^2^)	Male	28.81	0.12	29.93	1.47	29.85	0.95	26.50	0.54	0.2912
Body Mass Index (kg/m^2^)	Female	29.00	0.12	34.14	3.81	28.10	1.24	29.43	1.72	0.5816

a *Data are for adults, combined gender, aged 51–99 years-old (N = 182, Males = 74, Females = 108); Day 1 intakes; SE, standard error*.

## Discussion

Previous analyses using NHANES have reported associations with pasta consumption and nutrient intakes in adults, but not in younger populations. To our knowledge, the current NHANES analysis is the first to examine associations with pasta consumption, nutrient intakes and diet quality in children. Additionally, the present study is the first to consider tertile pasta consumption and associations with weight-related outcomes in adults. While the diet quality scores were low in both pasta consumers and non-consumers of pasta, the present analysis showed that pasta consumption was associated with a significantly higher diet quality score and greater intake of several 2015-DGA shortfall nutrients relative to non-consumers of pasta, including higher daily intakes of folate, iron, magnesium and dietary fiber. In addition, adults who consumed pasta had reduced added sugar intake. Specifically, pasta consumption was linked to 3.2 tsp eq lower added sugar per day when compared to those avoiding pasta foods. Significant associations were not seen when assessing total daily calories and sodium intake when comparing pasta consumers and non-consumers of pasta. In younger females only (i.e., 19–50 years of age), pasta intake across tertile ranges showed that pasta consumption was associated with reduced waist circumference, body weight, and BMI. Our previous work showed similar results for adults, such that body weight was significantly reduced in the pasta, cooked cereals, and rice dietary pattern relative to those not consuming any grain foods (10).

The current study results, using NHANES 2001–2012 data, are aligned with a recently completed NHANES 2009–2012 analysis (13), where researchers reported how various pasta dietary patterns in American adults can contribute differently to nutrient intakes and diet quality. Pasta and noodle consumers had better diet qualities relative to non-consumers of pasta and compared to consumers who primarily included macaroni and cheese dishes in their dietary patterns—the authors reported that macaroni and cheese pasta dishes tend to deliver greater total, and saturated fat, in addition to calories (13). Fulgoni and Bailey found that pasta/noodle consumers had higher dietary fiber intakes, ranging from 11.0 to 13.6% (1.89–2.35 g/day) (13). The current analysis showed higher fiber intakes in children (19.2% or 2.52 g/day) and adults (25% or 4.3 g/day), with percentage increases that may be reflective of the inclusion of additional years of NHANES datasets (i.e., 2001–2012 vs. 2009–2012). Likewise, data from the Moli-sani cohort in men and women ≥35 years-old found that pasta consumption was associated with better adhesion to the Mediterranean diet. The researchers reported that among food groups included in the Mediterranean diet, cooked tomatoes, onions, garlic, olive oil, cheese and rice were strongly correlated with pasta consumption in men and women. When considering data from the Italian Nutrition and Health Survey and the Moli-sani cohort, pasta consumption was also negatively associated with BMI, waist circumference, waist-to-hip ratio, and with a reduced prevalence of overweight and obesity (12). The current data showed that adult female pasta consumers in the 19–50 age group had better weight-related outcomes in comparison to non-consumers of pasta, while this association was not present in men. When reviewing the current dataset for pasta consumers, male pasta-eaters consumed greater amounts of calories (65%), alcohol drinks (71%), added sugar (57%), sodium (68%), and saturated fat (58%) daily relative to female pasta-eaters. These factors may have contributed to males having a less healthy overall diet, independent of pasta consumption. A recent discussion raised in a review by Gaesser (24) discussed the potential misconceptions around refined grains. While refined grain foods are one food component of the unhealthy Western dietary pattern—an eating pattern associated with increased chronic disease risk, including obesity. Gasser stated, “consequently, it is justified to ask is each food group in this dietary pattern independently contribute to increased risk of obesity, as has been described by the Dietary Guidelines Scientific Advisory Committee (25), or is it possible that the higher risk is not due to refined grain intake, but instead is a consequence of “guilt by association” with other foods in the dietary pattern?

The current pasta study did not differentiate between whole grain and refined/enriched grain pasta. However, as <5 percent of Americans meet established recommendations for whole grain intake, with the average American consuming <1 ounce-equivalent of whole grains per day (26, 27), the current study generated the assumption that the predominant type of pasta consumed in current dietary patterns is refined/enriched pasta. As such, the current NHANES data support that pasta consumption, including both whole and enriched pasta can provide value to the American diet, as pasta consumers had several nutrient intake benefits in addition to a better diet quality relative to non-consumers of pasta. Indeed, grain foods, including various pasta and pasta mixed dishes, inclusive of enrichment and/or fortification procedures, remain an important contributor of overall nutrition to the American diet. As of 1998, mandated folic acid fortification initiated by US Food and Drug Administration provided regulations for enriched bread, flour, cornmeal, rice and pasta (28). The Centers for Disease Control and Prevention (CDC) has stated that folic acid fortification of grain products has led to a 36% reduction in neural tube defects in a 10-year span and has prevented an estimated 10,000 neural tube defects in the US (29). Grains are an integral component of energy and nutrients in the 2015-DGA dietary patterns, such that at the 2000 calorie level, both the Healthy US- and Healthy Mediterranean-Style patterns propose 6-ounce equivalent (oz eq)/day servings of grains, with half of those servings sourced from whole grains, while the Healthy Vegetarian eating pattern recommends 6.5 oz eq/day servings of grains, with 3.5 oz eq/day from whole grains (5). The 2015-DGA encourages increased consumption of whole grains, while concurrently limiting and/or reducing refined and/or enriched grain foods in the diet. Simultaneously, whether through messaging from authoritative guidance and/or consumer and healthcare professional beliefs, processed foods are increasingly seen as foods to avoid and foods that are not compatible with currently recommended dietary patterns. However, not all processed foods may need to fall into the same classification as many processed foods, including grain food products are an important contributor of several nutrients, including shortfall nutrients identified by the 2015-DGA (30–35). Nutrient contribution of processed foods using data from NHANES 2003-2006 has also identified the nutritional importance enrichment and fortification practices play in the American population. Importantly, studies have also shown nutritional consequences linked to the removal of enriched/fortified foods from the diet. Specifically, when enriched and fortified foods are eliminated in the food supply, results show that large percentages of the population present inadequate intakes of several essential nutrients, including vitamins calcium, magnesium, iron, folate, thiamin, vitamin A, vitamin C, vitamin D, and vitamin E. In contrast, when nutrients from enrichment and fortification were added in modeling scenarios, the percentage of the population with inadequate intakes was meaningfully reduced for iron, folate, vitamin A, and vitamin D (34). Similarly, previous research in participants ≥2 years-old revealed substantial intake of essential nutrients result from the enrichment and fortification of select foods, suggesting that without this practice, nutrient intakes, and in particular nutrients of public health concern and/or shortfall nutrients, may be further exacerbated (35), further substantiating the positive role processed grain foods may play in public health nutrition. Thus, fortified and enriched foods, can be an integral component of nutrient-dense food patterns and can contribute imperative advantages to current and future dietary patterns via the contribution of dietary adequacy.

The present study acknowledges and contains limitations characteristic of NHANES research, which have been previously been reported. Data for energy and nutrient intakes, including values reported for diet quality, were obtained using 24-h dietary recalls, which rely on study participant memory. While validated procedures are used to collect the data, recalled information may be inaccurate and biased from misreporting, memory challenges and other potential measurement errors experienced in epidemiological research involving large datasets (36, 37). Further, the current evidence, being observational, cannot establish a causal link between the different grain foods patterns examined and improvements in diet quality, nutrient intakes and other health variables considered. The current data provide preliminary nutrition evidence and should not be extrapolated without further research completed. Additionally, the current study grouped surveys from different years and economic environments. For example. NHANES food and nutrient intake data from the early 2000's may be different from data during the economic crisis between 2008 and 2010. Also, the current analysis did not differentiate between whole grain and enriched grain pasta consumption, which was largely due to the small and insufficient sample size for whole grain pasta consumption. Future studies using NHANES may be able to differentiate differences in nutrient intake and diet quality between whole grain and enriched grain pasta food consumers, provided the sample sizes are sufficient. Nonetheless, a large strength of the current work stems from the use of NHANES, which is a large continuous survey that examines a nationally representative sample of about 5,000 individuals yearly by highly-trained medical personnel (18, 19). Additionally, we used numerous covariates in the present study for data adjustment purposes that limit potential confounding.

## Conclusions

The present study provides preliminary evidence that pasta can be included as part of a healthy diet in US children and adults, particularly as pasta intake may be related to food modifications that can increase the nutritional quality and variety for children and adults. The current analysis shows that pasta consumption was associated with a significantly better diet quality and greater intakes of several shortfall nutrients, including higher daily intakes of folate, iron, magnesium and dietary fiber relative to non-consumption of pasta. Pasta consumers also had lower daily intakes of saturated fat and added sugar compared to non-consumers of pasta with no differences observed in total daily calories and sodium intake. In particular, in adult females, aged 19–50 years of age, pasta consumption was associated with lower weight-related outcomes, which require further investigation. Maintaining recommended servings of pasta that fit within dietary recommendations to limit calories and monitor nutrients to limit and/or reduce (i.e., added sugar, saturated fat, and sodium) may have public health benefits. Future studies should explore associations between pasta and foods groups to encourage to determine if pasta serves as a vehicle for specific nutrient-dense, and routinely under-consumed food groups, including dairy, lean protein sources (i.e., lean meats, fish/seafood) and vegetables.

## Data Availability Statement

Publicly available datasets were analyzed in this study. This data can be found here: https://wwwn.cdc.gov/nchs/nhanes.

## Ethics Statement

The studies involving human participants were reviewed and approved by Research Ethics Review Board at the National Center for Health Statistics. Written informed consent to participate in this study was provided by the participants' legal guardian/next of kin.

## Author Contributions

YP, MPH, Ph.D. candidate, as SVP of Nutritional Strategies, collaborates with food, dietary supplements and beverage companies on various food, nutrition and regulatory affairs projects. YP was responsible for the intellectual conceptualization and design of the present analysis and the overall study. Additionally, YP was responsible for the gathering, analysis, and data interpretation in the current study and in the drafting of all versions of the manuscript.

## Conflict of Interest

YP is employed by Nutritional Strategies and has received honoraria and consulting fees from food and beverage companies and other commercial and non-profit entities with an interest in nutrition research.
